# Altered balance of excitatory and inhibitory learning in a genetically modified mouse model of glutamatergic dysfunction relevant to schizophrenia

**DOI:** 10.1038/s41598-017-01925-8

**Published:** 2017-05-11

**Authors:** David J. Sanderson, Aletheia Lee, Rolf Sprengel, Peter H. Seeburg, Paul J. Harrison, David M. Bannerman

**Affiliations:** 1 Department of Psychology, Durham University, Science Site, South Road, Durham, DH1 3LE UK; 2 Department of Experimental Psychology, University of Oxford, 9 South Parks Road, Oxford, OX1 3UD UK; 3 Max-Planck Institute of Medical Research, Department of Molecular Neurobiology, D-69120 Heidelberg, Jahnstrasse 29 Germany; 4 Department of Psychiatry, University of Oxford, and Oxford Health NHS Foundation Trust, Warneford Hospital, Oxford, OX3 7JX UK

## Abstract

The GluA1 AMPAR subunit (encoded by the *Gria1* gene) has been implicated in schizophrenia. *Gria1* knockout in mice results in recently experienced stimuli acquiring aberrantly high salience. This suggests that GluA1 may be important for learning that is sensitive to the temporal contiguity between events. To test this, mice were trained on a Pavlovian trace conditioning procedure in which the presentation of an auditory cue and food were separated by a temporal interval. Wild-type mice initially learnt, but with prolonged training came to withhold responding during the trace-conditioned cue, responding less than for another cue that was nonreinforced. *Gria1* knockout mice, in contrast, showed sustained performance over training, responding more to the trace-conditioned cue than the nonreinforced cue. Therefore, the trace-conditioned cue acquired inhibitory properties (signalling the absence of food) in wild-type mice, but *Gria1* deletion impaired the acquisition of inhibition, thus maintaining the stimulus as an excitatory predictor of food. Furthermore, when there was no trace both groups showed successful learning. These results suggest that cognitive abnormalities in disorders like schizophrenia in which gluatamatergic signalling is implicated may be caused by aberrant salience leading to a change in the nature of the information that is encoded.

## Introduction

It has been suggested that the positive, psychotic symptoms in disorders like schizophrenia, such as delusions, arise as a result of the aberrant or inappropriate assignment of salience to stimuli^1^. Aberrant salience is manifested as inappropriately high levels of attention and thus inappropriately determines behavior^1^ through altered learning and memory^2–4^.

While aberrant salience is classically associated with a hyper-dopaminergic phenotype^1^, it seems increasingly likely that this may represent a final common pathway, with primary disturbances elsewhere providing an initial trigger for behavioural changes^5^. There is increasing evidence that glutamatergic dysfunction and, particularly, consequent deficits in synaptic plasticity, might provide such an initial trigger^6, 7^. Recent genome-wide association studies have demonstrated a strong link between genes implicated in plasticity processes and glutamatergic neurotransmission, and the incidence of schizophrenia. For example, the locus containing *Gria1*, the gene that encodes the GluA1 (also known as GluR-A and GluR1) subunit of the AMPA subtype of glutamate receptor, shows genome-wide association with schizophrenia^8, 9^. Furthermore, reductions in GluA1 mRNA^10, 11^, GluA1 protein^12^, and AMPA receptor binding sites^13^ have been found in the hippocampus of schizophrenics. Mice in which the *Gria1* gene has been deleted (*Gria1* ^−/−^ mice) are useful for understanding the role of the GluA1 subunit in the mammalian brain. These mice exhibit a number of distinct phenotypes that include deficits in synaptic plasticity, particularly short-lasting forms of potentiation^14–17^. This is thought to reflect the important role of the GluA1 subunit in AMPAR trafficking and in the post-synaptic expression mechanisms underlying synaptic plasticity^18^. At a behavioral level, one finding in *Gria1* ^−/−^ mice is a deficit in a form of short-term memory that underlies short-term habituation. Thus, mice that lack *Gria1* fail to reduce attention to recently experienced, familiar stimuli^19–24^.

A predicted consequence of this change in attentional control caused by *Gria1* deletion is that it may lead to aberrant learning about the associations between stimuli, and thus, under some circumstances at least, qualitatively change the way that animals learn about their environment. For example, in normal animals the reduction in attention to recently experienced stimuli reduces the likelihood that associations will form between stimuli that, although presented in close temporal proximity, are not temporally contiguous. Therefore, normally there is a limited temporal window for learning to occur with the likelihood of excitatory associations forming between stimuli reducing as temporal separation increases. However, in *Gria1* ^−/−^ mice the prolonged attention that is paid to recently presented stimuli might increase the likelihood that temporally separated events will become associated, thus leading to aberrant learning^19^.

This prediction was tested by assessing the performance of *Gria1* ^−/−^ mice on an appetitive Pavlovian trace conditioning procedure in which there was temporal discontiguity between the conditioned stimulus (CS) and the outcome (food). Mice were trained on an auditory discrimination in which food was presented after one cue (CS+), but not after another (CS−). For some mice, food followed the CS+ immediately (no trace interval condition), but for others the interval between the CS+ and food delivery was either 4 or 8 s (trace CS+). Here we show that wild-type (WT) mice exhibit evidence for a temporally-controlled balance between excitatory and inhibitory associative learning during trace conditioning with the CS+ predicting the absence of food and the trace period indicating the occurrence of food. However, *Gria1* ^−/−^ mice demonstrate a pronounced shift towards excitatory learning, leading to the mis-attribution of salience to the CS+. Thus, while normal animals can effectively learn to use cues in their environment to predict not only when something will happen, but also when something is not going to happen based on temporal discontiguity, this ability is diminished in *Gria1* ^−/−^ mice.

## Results

### Training

We investigated the performance of *Gria1* ^−/−^ mice on an appetitive Pavlovian discrimination procedure in which food was presented after the reinforced cue (CS+), but not after the another, nonreinforced cue (CS−). Importantly, discrimination learning was assessed in both the presence and absence of temporal discontiguity between the reinforced cue (CS+) and the outcome (food) in separate groups of mice. The measure of responding was the time spent in the food magazine during the CS+ and CS− trials, prior to the presentation of food. Discrimination performance was assessed by calculating a discrimination ratio in which CS+ responding was expressed as a proportion of the total responses for both the CS+ and CS−. In addition to the analysis of the discrimination ratios, the raw rates of responding (from which the discrimination ratios were calculated) to the CS+ and CS− (expressed as percentage of the duration of the cue) were also analysed. Trace conditioning was initially compared to the no trace condition by collapsing across the 4 and 8 s trace interval conditions to form one trace group. Subsequent analyses that explored specific effects of genotype on trace conditioning, independent of the effects of genotype in the no trace condition, included the trace interval (4 or 8 s) as a factor in order to assess whether the effects of genotype were dependent on the trace interval.

#### Discrimination ratios

**Figure 1:**
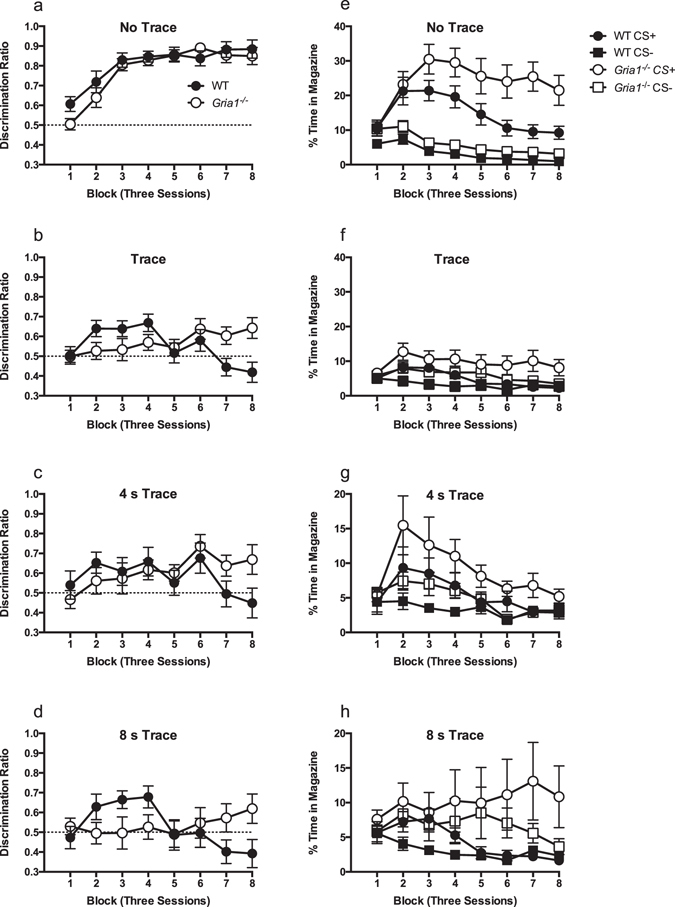
WT mice fail to show discrimination learning with prolonged training in the trace condition, but *Gria1* ^−/−^ mice show successful discrimination performance in both the trace and no trace conditions. Panels a–d show responding to the CS+ as a ratio of the total responding to the CS+ and CS−. Scores above 0.5 indicate greater responding to the CS+ than CS−, whereas scores below indicate greater responding to CS− than CS+. The dashed line indicates chance performance. Performance is shown across blocks of three sessions. Panels e–h show the raw rates of responding (time in the magazine as percentage of the cue duration) from which the discrimination ratios were calculated. Performance on the ‘no trace’ procedure is shown in panels a and e, whereas performance on the ‘trace’ procedure is shown in panels b and f. Panels c, d, g and h show the performance on the ‘trace’ procedure broken down by trace interval, with the 4 s interval in panels c and g and the 8 s interval in panels d and h. Error bars indicate ±S.E.M. Please note the difference in the scale of the y axes between panels e and f, and g and h, which have been adjusted to ease comparison of genotypes and trial types within the different trace interval conditions.

When there was no trace interval between the CS+ and food delivery both genotypes learned the discrimination at a similar rate (Fig. 1a). However, in the trace interval condition a difference between the genotypes emerged over training (Fig. 1b). Whereas *Gria1* ^−/−^ mice gradually acquired the discrimination in the trace condition as training continued, WT mice initially acquired the discrimination, but then performance changed such that at the end of training they were no longer making more responses to the CS+ than to the CS−, suggesting that the initial tendency to respond to the CS+ was inhibited with further training.

This was confirmed by a significant genotype by trace condition by block interaction (F(7,511) = 2.43, p = 0.033). The three-way interaction was then further investigated by conducting separate ANOVAs for the different trace conditions (no trace versus trace). For mice in the no trace condition there was a significant effect of block (F(7,168) = 26.37, p < 0.001), but no significant effect of genotype (F < 1, p > 0.3), and no significant genotype by block interaction (F(7,168) = 1.12, p > 0.3). In contrast, for mice in the trace condition there was a significant genotype by block interaction (F(7,343) = 5.82, p < 0.001). There was, however, no significant difference in performance of mice trained with either the 4 or 8 s trace interval, and the effect of trace interval did not significantly interact with genotype or block (p values > 0.1, see Fig. 1c and d). Simple main effects analysis of the genotype by block interaction revealed that *Gria1* ^−/−^ mice showed significantly superior discrimination than WT mice by the end of training, on both blocks 7 and 8 (smallest F(1,49) = 6.42, p = 0.015, block 7). Performance on the remaining blocks did not significantly differ between genotypes (largest F value, block 2, F(1,49) = 3.59, p = 0.064). Furthermore, whereas *Gria1* ^−/−^ mice showed a significant monotonic increase in performance over training (main effect of block, F(7,168) = 3.07, p = 0.005, significant linear trend, F(1,24) = 7.11, p = 0.014), WT mice showed a significant decline in performance over the later training blocks (main effect of block (F(7,165) = 5.09, p < 0.001, significant quadratic trend (F(1,25) = 12.76, p = 0.001)). Post-hoc analyses of the effect of block, using the Bonferroni correction, confirmed that the performance of WT mice on block 7 was significantly lower than on blocks 2 and 4 (p values < 0.05), and performance on block 8 was significantly lower than on blocks 2, 4 and 6 (p values < 0.05), demonstrating the decline in performance in the controls with extended training.

#### Time in the magazine

Analysis of the raw rates of responding revealed a similar pattern of results to the discrimination ratios (Fig. 1e and f), although it was clear the *Gria1* ^−/−^ mice responded at an overall higher level than compared to WT mice, resulting in the difference between the CS+ and CS− in the no trace condition being greater for *Gria1* ^−/−^ mice than WT mice. A 2 (trial type: CS+, CS−) by 2 (genotype) by 2 (trace condition: trace, no trace) by 8 (block) ANOVA revealed a significant four-way interaction (F(7,511) = 2.85, p = 0.038). The interaction was analysed by conducting separate ANOVAs for the no trace and trace conditions.

For the no trace condition (Fig. 1e) there was a significant three-way interaction of factors (F(7,168) = 3.80, p = 0.018). This three-way interaction was further analysed by conducting separate ANOVAs for each block. There was a significant trial type by genotype interaction on the first block (F(1,24) = 5.22, p = 0.032) reflecting that WT mice responded more to the CS+ than CS− (F(1,24) = 12.45, p = 0.002), but not this was not true for *Gria1* ^−/−^ mice (F < 1, p > 0.8). There were also a significant interaction between trial type and genotype on blocks 6–8 (smallest F(1,24) = 5.09, p = 0.033, block 8). Simple main effects analysis demonstrated that this was due to the effect of trial type being greater for *Gria1* ^−/−^ mice (smallest F(1,24) = 31.29, p < 0.001, block 8) than for WT mice (smallest F(1,24) = 7.40, p = 0.012, block 8). There was no significant interaction between factors on blocks 2–5 (largest F(1,24) = 2.38, p = 0.14, block 5).

For the trace condition (Fig. 1f) there was also a significant trial type by genotype by block interaction (F(7,329) = 2.90, p = 0.038). The effect of the duration of the trace interval (4 or 8 s) was not significant and did not interact with other factors (largest p value = 0.094; see Fig. 1g and h).The trial type by genotype by block interaction was analysed by conducting separate ANOVAs for each block. There was a significant trial type by genotype interaction on blocks 7 and 8 (smallest F(1,47) = 6.00, p = 0.018, block 8). Simple main effects analysis demonstrated this was due to *Gria1* ^−/−^ mice showing successful discrimination between the trial types (smallest F(1,47) = 9.62, p = 0.003, block 8), but WT mice did not (F values < 1, p values > 0.70). In addition, while the genotypes did not significantly differ in the extent of responding to the CS− (largest F(1,47) = 1.55, p = 0.22, block 7), *Gria1* ^−/−^ mice showed greater responding to the CS+ than WT mice (smallest F(1,47) = 5.84, p = 0.020, block 8). There was no significant trial type by genotype interaction on the blocks 1–6 (largest F(1,47) = 1.63, p = 0.21, block 6). Mice did, however, respond significantly more to the CS+ than to CS− on blocks 2, 3, 4 and 6 (smallest F(1,47) = 8.90, p = 0.005, block 6), but not on blocks 1 and 5 (largest F(1,47) = 3.35, p = 0.07, block 5).

### Responding during the trace interval

#### Discrimination ratios

**Figure 2:**
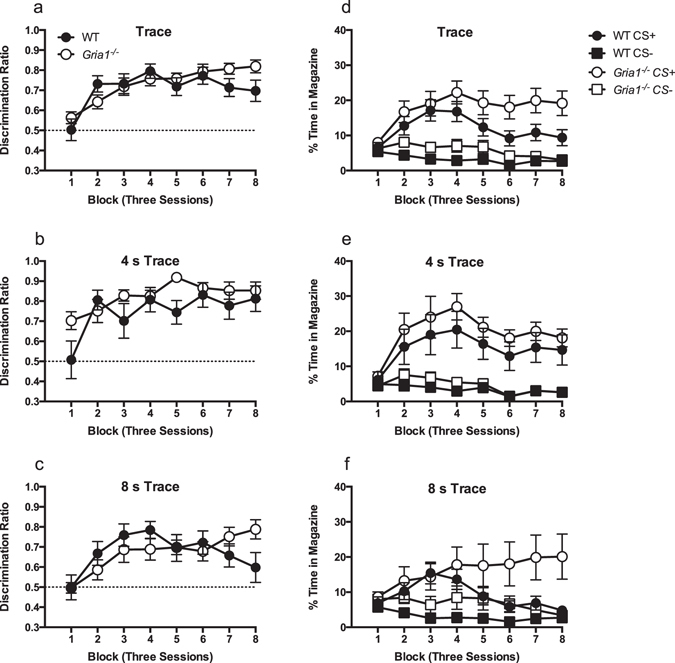
*Gria1* ^−/−^ and WT mice show similar, successful acquisition of discrimination performance during the trace interval. Panels a–c show responding in the trace interval between the termination of the CS+ and presentation of food as a ratio of the total responding to the CS+ trace interval and the equivalent period after the CS−. Scores above 0.5 indicate greater responding to the CS+ than CS−, whereas scores below indicate greater responding to CS− than CS+. The dashed line indicates chance performance. Performance is shown across blocks of three sessions. Panels d–f show the raw rates of responding (time in magazine as a percentage of the trace interval) from which the discrimination ratios were calculated. Performance during the trace interval is shown in panels a and d, collapsed across trace interval (4 and 8 s). The performance during the trace interval for the 4 and 8 s conditions is shown separately in panels b and e, and c and f, respectively. Error bars indicate ±S.E.M.

The observation that wild-type mice did not demonstrate any discrimination between the CS+ and CS− cues by the end of training was somewhat surprising. However, it is possible that mice in the trace conditions will learn that food delivery occurs at a time point after the end of the auditory cue (e.g., ref. 25), and will come to selectively respond in the trace interval rather than the CS. Therefore, for mice in the trace conditions (4 or 8 s), responding throughout the trace interval (i.e., between the end of the auditory stimulus and the delivery of the reinforcement) was also analysed. As expected, performance increased as training proceeded such that both WT and *Gria1* ^−/−^ mice were making proportionally greater responses during the trace interval for the CS+ than for the equivalent period of time after the CS− (see Fig. 2a; effect of block, F(7,13) = 13.17, p < 0.001). In the last two blocks *Gria1* ^−/−^ mice showed a slightly higher level of discrimination than WT mice, however the genotype by block interaction failed to reach significance (F(7,329) = 2.31, p = 0.054), Both genotypes, however, showed an increase in performance over blocks (WT, F(7,168) = 5.80, p < 0.001; *Gria1* ^−/−^, F(7,161) = 12.29, p < 0.001). Furthermore, despite WT mice failing to discriminate between the CS+ and CS− during the presentation of the auditory cues on blocks 7 and 8 (see Results, Training), they did show successful discrimination during the trace period for those blocks with performance significantly above chance (smallest t(25) = 3.68, p = 0.001, block 8). This was true for *Gria1* ^−/−^, mice as well (smallest t(24) = 9.83, p < 0.001, block 8). Overall, discrimination ratios were significantly higher for the 4 s trace interval than for the 8 s interval (F(1,47) = 7.74, p = 0.008, see Fig. 2b and c), demonstrating better discrimination learning for the shorter trace interval. There were no other significant main effects of interactions (p values > 0.2).

#### Time in the magazine

Analysis of the raw rates of responding revealed a similar pattern of results to the discrimination ratios, although it was again clear that *Gria1* ^−/−^ mice responded at a greater level than WT mice (F(1,47) = 4.35, p = 0.042; see Fig. 2d). Time in the magazine, during the trace interval periods, increased for CS+ trials over training, but not for CS− trials (trial type by block interaction: F(7,329) = 11.75, p < 0.001). There was a significant trial type by trace interval interaction (F(1,47) = 4.82, p = 0.033), reflecting that the difference between the CS+ and CS− was greater in the 4 s condition than in 8 s condition (Fig. 2e and f). There were no other significant interactions (p values > 0.06).

### Responding over the duration of the CS

#### Discrimination ratios

The initial analyses of responding during the auditory cues demonstrated that in the trace condition, the WT and *GluA1* ^−/−^ mice differed in the final two blocks (blocks 7 and 8, Fig. 1b) of training. Whereas WT mice failed to show greater responding to the CS+ than to the CS− by the end of training, *Gria1* ^−/−^ mice showed successful discrimination, exhibiting greater responding to the CS+ than to the CS− (as indicated by the discrimination ratios). In contrast, during the trace interval both WT and *Gria1* ^−/−^ mice showed successful discrimination (see Fig. 2a). This pattern of performance in the WT mice suggests that they were withholding responding during the CS+ and then selectively responding in the trace interval. *Gria1* ^−/−^ mice, however, were responding during both periods.

**Figure 3:**
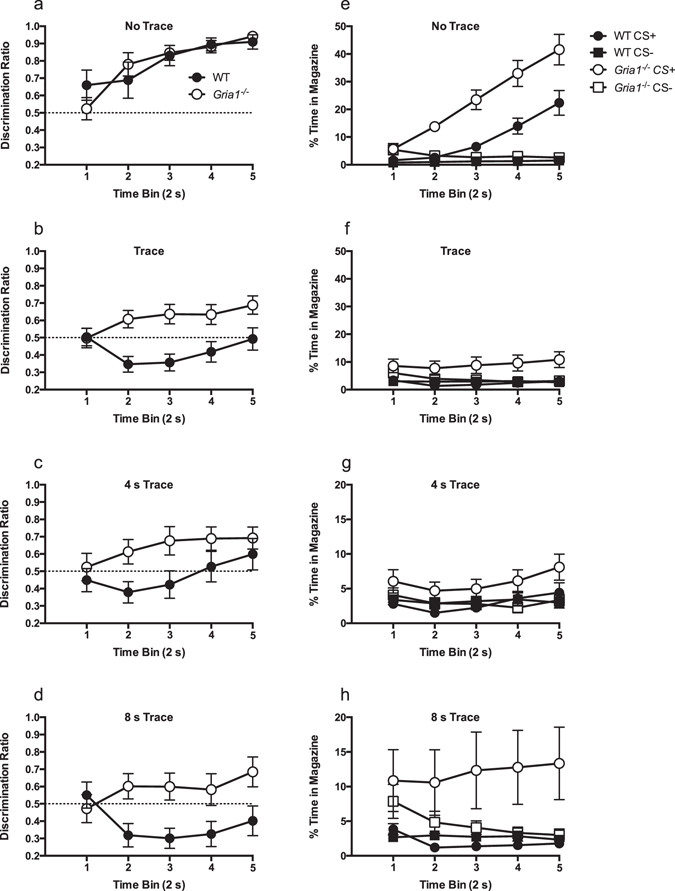
WT mice inhibit responding during the trace CS+, relative to baseline, whereas *Gria1* ^−/−^ mice increase CS+ responding relative to baseline during the course of the CS. Discrimination performance across the 10 s duration of the conditioned stimuli is shown in 2 s bins for the final two blocks (see Fig. 1) of training. Panels a–d show responding to the CS+ as a ratio of the total responding to the CS+ and CS−. Scores above 0.5 indicate greater responding to the CS+ than CS−, whereas scores below indicate greater responding to CS− than CS+. The dashed line indicates chance performance. Panels e–h show the raw rates of responding (time in magazine as a percentage of the time bin) from which the discrimination ratios were calculated. Panels a and e shows the performance on the ‘no trace’ condition and panels b and f show the ‘trace’ condition collapsed across trace interval (4 and 8 s). The performance on the ‘trace’ condition with the 4 and 8 s intervals is shown separately in panels c and g and d and f, respectively. Error bars indicate ±S.E.M. Please note the difference in the scale of the y axes between panels e and f, and g and h, which have been adjusted to ease comparison of genotypes and trial types within the different trace interval conditions.

In order to examine more closely the pattern of responding during the time course of the CS+ and CS− cues at the end of training, across blocks 7 and 8, responding during the ten second CS presentations was divided into five 2 s bins (see Fig. 3). For comparison, separate analyses were conducted for the no trace condition (Fig. 3a) as well as the trace conditions (Fig. 3b).

For the no trace interval condition (see Fig. 3a), WT and *Gria1* ^−/−^ mice showed a monotonic increase in discrimination performance over the course of the CS duration such that they were discriminating best at the end of the cues (effect of time bin, F(4,96) = 16.24, p < 0.001). There was no significant effect of genotype (F < 1), nor genotype by time bin interaction (F(4,96) = 1.72, p > 0.1).

In the trace condition, discrimination performance in WT mice was at chance level at the beginning of the stimulus presentation, indicating equivalent responding to the CS+ and CS− (Fig. 3b). However, during the middle of the CS duration (bins 2–4) performance actually went below chance level, indicating that the CS+ elicited less responding than the CS−. By the last 2 s bin of the CS duration performance had returned to chance level. In contrast, *Gria1* ^−/−^ mice showed a gradual increase in discrimination performance over the duration of the CS, indicating that relative responding to the CS+, as compared to the CS−, increased progressively over time.

This pattern was confirmed by a significant genotype by time bin interaction (F(4,188) = 4.29, p = 0.007). Simple main effects analysis of this interaction showed that there was an effect of genotype in time bins 2–5 (smallest F(1,47) = 5.12, p = 0.028, block 5), but not for time bin 1 (F < 1, p > 0.9). WT mice showed a significant effect of time bin (F(4,96) = 3.41, p = 0.033), with a significant quadratic trend (F(1,24) = 24.96, p < 0.001), demonstrating that performance went below chance level and then returned to chance. Post-hoc analyses of the effect time bin, using the Bonferroni correction showed that performance in time bin 2 was significantly lower than time bin 1 (p = 0.026), but all other comparisons were not significant (p values > 0.07). One-sample t-tests, comparing the discrimination ratios against chance (0.5), showed that WT performance was significantly below chance on time bins 2 and 3 (smallest t(25) = 2.93, p = 0.007, time bin 3). In contrast to the performance of the WT mice, *Gria1* ^−/−^ mice showed a significant effect of time bin (F(4,92) = 3.84, p = 0.006) with a significant linear trend (F(1,23) = 8.05, p = 0.009), demonstrating the gradual increase in discrimination performance across the CS duration. One-sample t-tests showed that performance was significantly above chance on time bins 2–5 (smallest t(24) = 2.15, p = 0.042, time bin 2), but not on time bin 1 (t < 1, p > 0.9). The effect of trace interval (4 s or 8 s) was not significant (F(1,47) = 1.49, p > 0.2) and did not significantly interact with other factors (p values > 0.1, see 3c and 3d).

#### Time in the magazine

Analysis of the raw rates of responding revealed a similar pattern to the discrimination ratios, although, once again, it was clear that *Gria1* ^−/−^ mice responded at an overall higher rate compared to WT mice. For the no trace condition a 2 (genotype) by 2 (trial type) by 5 (time bin) ANOVA revealed a significant three-way interaction (F(4,96) = 6.27, p = 0.01), reflecting that as time progressed the difference in responding to the CS+ and CS− was greater in *Gria1* ^−/−^ mice than compared to WT mice (see Fig. 3e).

For the trace condition (see Fig. 3f) there was a significant three-way interaction between trial type, bin and genotype (F(1,47) = 4.30, p 0.025). The effect of trace interval (4 or 8 s) was not significant and did not significantly interact with other factors (p values > 0.060, see Fig. 3g and h). In order to analyse the three-way interaction separate ANOVAs for each genotype were conducted. For WT mice there was a significant trial type by bin interaction (F(4,96) = 4.37, p = 0.014). Simple main effects analysis showed that WT mice responded at a significantly lower level to the CS+ than to the CS− on bins 2 and 3 (smallest F(1,24) = 4.73, p = 0.04). There was no significant difference on bins 1, 4 and 5 (F values < 1, p values > 0.50). There was a significant effect of bin for the CS+ (F(4,96) = 4.41, p = 0.022) with significantly lower responding on bins 2 and 3 compared to bin 1 (largest p value = 0.025, Bonferroni corrected; all other comparisons p > 0.06). The effect of bin for the CS− was not significant (F < 1, p = 0.74).

For *Gria1* ^−/−^ mice there was a significant trial type by bin interaction (F(4,92) = 5.03, p = 0.023). Simple main effects analysis showed *Gria1* ^−/−^ mice responded more to the CS+ than to the CS− on bins 4 and 5 (smallest F(1,23) = 4.23, p = 0.021), but not on bins 1, 2 and 3 (largest F(1,23) = 4.23, p = 0.051). While the effect of bin was not significant for the CS+ (F(4,92) = 3.37, p = 0.055), there was a significant effect for the CS− (F(4,92) = 5.52, p = 0.016). All pairwise comparisons between bins, however, were not significant (p values > 0.10, Bonferroni corrected).

Thus, in summary, wild-type mice learned to inhibit responding to the CS+ cue during its presentation and then subsequently respond in the trace interval. In contrast, *Gria1* ^−/−^ mice responded continually throughout the cue and the trace interval.

## Discussion

In order to investigate temporal aspects of associative learning and aberrant salience in a mouse model of glutamatergic dysfunction that is relevant to schizophrenia, we examined trace conditioning in *Gria1* ^−/−^ mice. WT and *Gria1* ^−/−^ mice showed similar acquisition, in terms of the accuracy of responding (as indicated by the discrimination ratios) of an appetitively motivated, auditory discrimination task when there was no trace interval. However, when there was a trace interval between the CS+ and food delivery, WT and *Gria1* ^−/−^ mice exhibited markedly different patterns of performance. WT mice initially acquired the discrimination, but notably as training progressed, performance changed such that WT mice no longer exhibited greater responding to the CS+ than the CS− by the end of training. In contrast, *Gria1* ^−/−^ mice showed a progressive increase in discrimination performance across training, such that responding to the CS+ remained greater than for the CS−.

The absence of discrimination performance between the auditory cues in the WT mice in the trace condition at the end of training was in stark contrast to both their performance earlier in training, and their successful discrimination performance when measured during the subsequent trace interval, which was similar to that shown by *Gria1* ^−/−^ mice. Further analysis of the pattern of discrimination performance during the time course of the auditory cues in the final blocks of testing demonstrated that WT mice were actively inhibiting responding to the CS+ (i.e., they were actually responding at a higher level to the nonreinforced CS− than to the reinforced CS+). In contrast, *Gria1* ^−/−^ mice showed a monotonic relative increase in responding the CS+ (compared to the CS−) over the course of the auditory cues. Collectively, these results demonstrate that while *Gria1* ^−/−^ mice showed greater discrimination performance than WT mice in the trace conditioning procedure, this enhanced performance reflects the failure of *Gria1* ^−/−^ mice to learn to inhibit responding to the trace conditioned stimulus with continued training.

The fact that WT mice in the trace condition came, with prolonged training, to restrict responding to time points close to the presentation of the reinforcement (i.e., during the trace interval) suggests that they timed the occurrence of reinforcement more accurately than *Gria1* ^−/−^ mice. A timing account, however, cannot explain why WT mice withheld responding during CS+ in the trace condition such that responding was lower than for the CS−. Timing accounts propose that conditioned responding is inversely proportional to the expected time of the next presentation of reinforcement, such that as the expected time decreases, responding increases^26^. In the trace condition the time between the onset of the CS+ and reinforcement was far shorter than that between the CS− and the next presentation of reinforcement that would occur a number of minutes later. Therefore, while timing accounts may predict that accurate timing may lead to responding during the CS+, in the trace condition, being no greater than for the CS−, it would not be expected that CS+ responding would be lower than CS− responding.

The active withholding of responding by WT mice to the CS+ in the trace condition suggests that, although the CS+ was initially an excitatory predictor of food (eliciting anticipatory magazine responding), as training progressed the CS+ became an inhibitory signal, predicting the absence of food. Therefore, WT mice avoided the food magazine during the CS+ presentation, in a manner that is similar to that found with conditioned inhibitor cues that signal the absence of otherwise expected events^27^. During the trace interval, however, WT mice did make anticipatory magazine responses suggesting that the CS trace memory, or the offset of the CS, rather than the CS itself, was a signal for the occurrence of food. The ability of a trace conditioned stimulus to become a conditioned inhibitor is not unprecedented^28–31^. Furthermore, it suggests that the temporal discontiguity in the trace conditioning procedure may lead to not only a weakening of excitatory associative learning, but also an active strengthening of inhibitory learning. This is similar to the inhibition of delay effect, in which conditioned responding declines to the early portions of a conditioned stimulus as training progresses^32–34^.

According to many trial-based models of learning such as the Rescorla-Wagner model^35^, inhibition occurs because a cue is paired with the violation of an expected outcome (i.e., negative prediction error). For example, in a discrimination procedure a nonreinforced CS− may become inhibitory because the context is a predictor of the unconditioned stimulus (US), but the US never occurs in the presence of the compound of the context and the CS−^36^. However, negative prediction cannot explain why, in the present experiment, the CS+ in the trace condition came to be a conditioned inhibitor for WT mice, eliciting weaker responding than the CS−, because, all other things being equal, the negative prediction error would be the same for the trace CS+ and CS−.

The only difference in the trace condition between the CS+ and the CS− was the temporal proximity to the US. Therefore, a time-dependent process must have caused inhibition. It has been suggested that the temporal dynamics of short-term memory decay is a key determinant of the formation of inhibitory associations between stimuli^37–40^. Specifically, the mnemonic representations of currently experienced stimuli are processed in a primary short-term memory state (akin to processing that is the focus of attention), whereas the representations of recently experienced stimuli are processed in a different, secondary short-term memory state (akin to processing that is in the periphery of attention^40^). Processing of representations in the same short-term memory state will lead to excitatory associations that result in conditioned responding, but concurrent processing of representations in different short-term memory states leads to inhibitory associations that result in stimuli acquiring the ability to inhibit the retrieval of memories^41, 42^. In trace conditioning, the temporal discontiguity between the CS and the outcome (US), will lead to the outcome being processed in the primary short-term memory state while the CS representation is now in the secondary short-term memory state, and, therefore, inhibitory learning will occur and the CS will become a conditioned inhibitor.

This theory provides an account of the performance of WT mice observed at the end of training in the present study. However, it does not anticipate that trace conditioning will initially be excitatory and then subsequently inhibitory. The fact that learning was initially excitatory suggests that the representation of the CS+ had only partially transferred to the secondary short-term memory state by the time that food was presented, and therefore, the CS representation received sufficient processing in the primary short-term memory state to allow initially some excitatory learning. It is possible that the overall switch from excitation to inhibition reflects that inhibitory learning is often proposed to be slower than excitatory learning^35, 40, 43^. However, it would also have to be assumed that inhibitory learning can achieve an overall higher level than excitatory learning such that the subsequent acquisition of inhibition can outweigh the initial excitatory learning.

We have previously demonstrated that *Gria1* deletion impairs short-term habituation, suggesting that it retards the rate that representations of recently presented stimuli transfer from the primary short-term memory state to the secondary short-term memory state (i.e., mnemonic representations of recently presented stimuli remain the focus of attention for longer in *Gria1* ^−/−^ mice^19–22^). Applying this analysis of *Gria1* deletion to the current results can explain the qualitatively different forms of learning displayed by the two genotypes. A reduction in the rate of transfer from the primary to secondary short-term memory state in *Gria1* ^−/−^ mice will result in weaker secondary state activity at the time that food is presented. The reduced secondary state activation will weaken the ability of the CS+ in the trace condition to become a conditioned inhibitor. Therefore, the enhanced responding to the CS+ in the trace condition in *Gria1* ^−/−^ mice likely reflects a consequence of failing to learn an inhibitory association between the CS and food. Overall, a reduction in the rate of transfer from the primary to secondary short-term memory state will lead to an increase in excitatory learning due to greater concurrent primary memory state processing of events that occur in relatively close temporal proximity. This may provide an account of the general increase in magazine activity seen in *Gria1* ^−/−^ mice, and particularly the increased responding to the CS+ in the no trace condition. The increase in responding, however, did not necessarily result in superior accuracy of learning, because the discrimination ratios in the no trace condition did not differ. Furthermore, it is not possible to rule out potential general increases in activity^44^.

The present results demonstrate that WT and *Gria1* ^−/−^ mice form qualitatively different associations when tested under a trace conditioning procedure. We have previously shown that *Gria1* ^−/−^ mice can form long-term memories under conditions that WT mice do not^19^. We have argued that this aberrant learning in *Gria1* ^−/−^ mice is due to stimuli maintaining an aberrantly high level of salience as a result of deficits in short-term habituation. Whereas WT mice quickly reduce attention to stimuli as they are experienced, *Gria1* ^−/−^ mice are less able to reduce their attention to recently experienced stimuli in the appropriate manner. The aberrant learning caused by abnormally high levels of attention to recently experienced stimuli provides a link between *Gria1* and abnormal psychological processes implicated in schizophrenia. Here we show that deficits in attention resulting from *Gria1* deletion can lead, not only to learning occurring in situations where no learning would occur in wild-types, but also to a qualitative change in the nature of learning caused by the balance between excitatory and inhibitory learning. Importantly, an analysis of GluA1 function in terms of regulating the rate of decay of short-term memories and attentional processes, provides a psychological mechanism for determining how aberrant salience can lead to the abnormal formation of associations between events, of particular relevance to delusion formation and perhaps other cognitive abnormalities in disorders like schizophrenia in which altered glutamatergic signaling is implicated.

## Methods

### Subjects

Experimentally naïve, age-matched, littermate, male and female, wild-type and *Gria1* ^−/−^ mice, bred in the Department of Experimental Psychology, University of Oxford, served as subjects (see ref. 17 for details of genetic construction, breeding and subsequent genotyping). Mice were caged in groups of 2–6, in a temperature controlled housing room on a 12 h light/dark cycle (0700–1900). All testing was conducted during the light period. Mice were approximately 6 months old at the start of testing. Mice were initially allowed free access to food, but prior to training the weights of the mice were reduced, by receiving a restricted diet, and then subsequently maintained at 85% of their free-feeding weight. Throughout testing mice had ad libitum access to water in their home cages. All procedures were in accordance with the United Kingdom Animals Scientific Procedures Act (1986) and were approved by the UK Home Office; under project license number PPL 30/2561.

### Apparatus

Eight identical operant chambers (15.9 × 14.0 × 12.7 cm; ENV-307A, Med Associates), enclosed in sound-attenuating cubicles (ENV-022MD, Med Associates), controlled by Med-PC IV software were used. The front and back walls and the ceiling of each chamber were made from clear Perspex and the sidewalls were made from aluminium. The floor was a grid of stainless steel rods (0.32 cm diameter) each separated by 0.79 cm. Sucrose pellets (20 mg TestDiet, ETH) could be dispensed into a magazine (2.9 × 2.5 × 1.9 cm; ENV-303M, Med Associates) located in the centre of one of the sidewalls. Breaks in an infrared beam (ENV-303HDM, Med Associates) across the bottom of the entrance to the magazine were used to detect head entries into the magazine. White noise and a pure tone (3 kHz), each at 75 dB (~10 dB above background noise), generated by an audio generator (ANL-926, Med Associates) could be emitted from a speaker (ENV-324M, Med Associates) located at the top right corner of the wall opposite the magazine. A fan positioned in the left corner above the magazine (ENV-025AC, Med Associates) was turned on during sessions.

### Procedure

Mice received two sessions of magazine training, one per day, prior to the start of discrimination training. Within a session, ten pellets were dispensed into the magazine on a variable time schedule of 260 s (range = 13–859 s, based on Fleshler-Hoffman distribution^45^). Twenty-four hours after the last magazine training session mice received 24 sessions of discrimination training, one per day. Each session consisted of 10 presentations of the CS+ and 10 presentations of the CS−. For approximately half the mice, within each genotype, sex and trace condition (i.e. no trace, 4 or 8 s trace conditions), the CS+ was a 10 s presentation of white noise and the CS− was a 10 s presentation of pure tone. For the remaining mice, the allocation of stimuli to trial types was reversed. The trial types were presented in a random order with the constraint that there were an equal number of CS+ and CS− trials every block of four trials. Each trial was separated by a fixed interval of 120 s (CS offset to CS onset).

Mice were allocated to one of three conditions: 0 s (no trace) condition (WT: female, N = 7, male, N = 7; *Gria1* ^−/−^: female, N = 6, male, N = 6), 4 s trace condition (WT: female, N = 6, male, N = 6; *Gria1* ^−/−^: female, N = 6, male, N = 6) or 8 s trace condition (WT: female, N = 7, male, N = 7; *Gria1* ^−/−^: female, N = 6, male, N = 7) trace interval. For mice in the no trace condition a food pellet was dispensed at the offset of the CS+. For mice in the 4 s and 8 s conditions, the interval between offset of the CS+ and food pellet delivery was 4 s and 8 s, respectively. No food pellets were presented after the CS−.

### Measures and Statistical Analyses

The amount of time that a mouse spent in the magazine during the CS+ was used as the measure of conditioned responding and was expressed as a ratio of the total amount of time spent in the magazine during the CS+ and CS− (discrimination ratio). Discrimination ratios greater than 0.5 indicate greater responding to the CS+ than CS−, whereas vice versa for ratios below 0.5. A ratio of 0.5 indicates equal responding to both CSs. Discrimination ratios were also calculated for responses made during the trace interval (between the end of the auditory cue and the delivery of the sucrose pellet) and for the equivalent period of time after the termination of the CS−. In addition to the analysis of discrimination ratios the rates of responding (time in the magazine) for the CS+ and CS− (from which the discrimination ratios were calculated) were also analysed. Time in the magazine was expressed as a percentage of the available time.

Discrimination ratios and raw rates of responding were analysed using multifactorial ANOVAs. Significant interactions were analysed using simple main effects analysis, using the pooled error term from the original ANOVA, and separate ANOVAs for within-subjects factors with more than two levels. The Greenhouse-Geisser correction was used in any instances of violations of sphericity. Initial analyses examined the effect of temporal discontiguity by comparing the mice trained on the trace conditioning procedure, collapsed across trace interval (4 and 8 s), with mice trained on the “no trace” procedure. Therefore, the results of the 4 and 8 s interval conditions were pooled to form one “trace” condition group. When effects of genotype were found on trace conditioning subsequent analyses of these effects included the trace interval (4 or 8 s) as a between-subjects factor in order to examine if genotype effects interacted with trace interval.
